# Reengineering Vital Registration and Statistics Systems [Response to Letter]

**Published:** 2004-12-15

**Authors:** Charles J Rothwell

**Affiliations:** National Center for Health Statistics, Centers for Disease Control and Prevention, Hyattsville, Md

## In Reply:

Dr Nasseri has registered valid concerns (1) about reengineering vital registration and statistics systems for the United States (2), many of which I share. Certainly the move to state-of-the-art information technology will not be the only key to rejuvenating the vital statistics system. We must not miss this opportunity to improve both the quality and the content of the information provided by these reengineered systems. The expanded data set derived from the new standard certificates, once implemented by the new systems, will improve data content extensively. These certificates are available on the Internet (3). Considerable work lies ahead in finding innovative ways to improve data quality for both new and old data items.

Dr Nasseri also suggests a number of demographic indicators for the death certificate that would have analytic and research value, including the place (country) of birth of the decedents' parents and occupation. Some of these items are of great potential value. The panel of experts (4) that recommended to the Secretary of the Department of Health and Human Services the new and revised data items to be included in the new standard certificates  consisted of a broad range of data providers and users as well as state vital registrars and statisticians. Three questions guided the panel's decisions on maintaining or revising old items and adding new items: 1) Is the item needed for legal, research, statistical, or public health programs? 2) Is the item collectable with reasonable completeness and accuracy? 3) Is the vital statistics system the best source for this information? The panel worked exceedingly hard and successfully to balance important public health needs with the most appropriate mechanism to collect information with the concern that the collection of this information not be an unreasonable burden for respondents. As Dr Nasseri notes, state registration and health officials are free to add items to their own vital records systems when they believe these additional questions are needed for state purposes. With an advanced and flexible information technology infrastructure in place, such additions become more practical at the state level and could in the future serve as a method to effectively pilot test items for inclusion in new national standards. State data users and providers need to be very involved in implementing and updating these systems in their own state.

Information on place of birth of the decedent is requested on the death certificate, and information on place of birth of parents is requested on the birth certificate. Specifications developed for coding and editing data from the 2003 revisions of the U.S. Standard Certificates include detailed information on country of birth, with an extended appendix of possible entries included in the electronic systems (5,6). The National Center for Health Statistics has every expectation that the detailed information on country of birth will be available in the national data sets based on the 2003 revisions of the standard certificates.

Information on educational attainment has changed in vital records. For many years, standard birth and death certificates have included items asking for number of years of schooling completed. Information from these items was used in combination with census population estimates based on similar questions to compute population-based fertility and mortality rates by educational attainment. However, the 2000 census modified the educational attainment question to collect information on the highest degree attained (e.g., high school diploma, associate's degree, master's degree). The change from years of school completed to degree attained was made because years of schooling completed are less synchronized than they used to be with degree attained, especially for college years. The focus on highest degree attained provides more consistent and uniform information. Because of the change by the U.S. Census Bureau, the expert panel recommended a similar change for the U.S. Standard Certificates so that the numerators for fertility and mortality rates (births and deaths) would be compatible with the denominators (4); the 2003 revisions of the standard certificates reflect this change.
